# Obituary

**Published:** 1909-12-04

**Authors:** 


### Obituary.

We have to record the death of Mr. Frederick Stocks as the result of an accident. Mr. Stocks, who was 65 years of age, took his M.R.C.S. in 1866, having studied at St. Thomas's, where he held the post of resident accoucheur. At one time he was assistant medical officer to the County Asylum at Rainhill. At the inquest held on November 30, the jury returned a verdict to the effect that Mr. Stocks was killed on the line at Walton-on-Thames, but that there was not sufficient evidence to prove how he came there.

The death is announced of Mr. Mark Farrant, of Exeter, at the age of thirty-eight. Mr. Farrant, who won an entrance scholarship at Westminster Hospital, took his diploma in 1893, and held many appointments as medical officer of health. He was a Fellow of the Society of Medical Officers of Health, and contributed "Notes on a Small Outbreak of Scarlet Fever" to *Public Health*.

